# Acute macular neuroretinopathy and COVID-19 or SARS-CoV-2 infection: case report and literature review

**DOI:** 10.3389/fmed.2024.1267392

**Published:** 2024-02-07

**Authors:** Xing Wang, Peng Wang, Jing Lu, Huan Ju, Hao Xie, Hui Peng

**Affiliations:** ^1^Department of Clinical Medicine, Chongqing Medical University, Chongqing, China; ^2^Chongqing Key Laboratory of Ophthalmology, Department of Ophthalmology, Chongqing Eye Institute, The First Affiliated Hospital of Chongqing Medical University, Chongqing, China

**Keywords:** COVID-19, SARS-CoV-2, acute macular neuroretinopathy, paracentral scotomas, corticosteroids

## Abstract

**Purpose:**

To describe a case of acute macular neuroretinopathy (AMN) associated with COVID-19 infection and a related literature review.

**Methods:**

A case from the First Affiliated Hospital of Chongqing Medical University was reported that could be linked to COVID-19 or SARS-CoV-2 infection. We performed a comprehensive search on PubMed, retrieving articles containing information on AMN after COVID-19 or SARS-CoV-2 infection. The key words used were ‘COVID-19’, ‘SARS-CoV-2’, ‘ophthalmic manifestations’, ‘acute macular neuroretinopathy’, and ‘paracentral scotomas’. The relevant data were extracted, charted, consolidated, and evaluated. Moreover, manual exploration of the reference lists of pertinent articles was carried out.

**Results:**

We describe the case of a 30-year-old young woman who developed bilateral AMN one day after being infected with COVID-19 or SARS-CoV-2. She had severe visual impairment (20/2000 OD and 20/32 OS), and her vision recovered after taking oral corticosteroids. After reviewing the literature, we summarized 16 relevant reports and found that symptoms of AMN tend to arise 1 day to 1 month after COVID-19 or SARS-CoV-2 infection. Contraceptive pills and other risk factors should be avoided to reduce the risk of adverse outcomes. Oral prednisone may be an effective treatment for those experiencing important vision loss.

**Conclusion:**

Symptoms of AMN can arise 1 day to 1 month after COVID-19 or SARS-CoV-2 infection. Ophthalmologists should remain vigilant about this disease, notably because patient characteristics may deviate from the norm.

## Introduction

The 2019 coronavirus disease (COVID-19) pandemic has been a substantial public health concern (1). With the continuous mutation of the virus (2) and the expansion of the scope of infection, an increasing number of eye lesions are caused. The clinical manifestations of novel coronavirus eye disease are diverse and lack specificity. Symptoms include many aspects, such as ocular inflammatory reaction disease (3–6), vascular disease (7, 8), and neurological disease (9, 10). Acute neuroretinopathy following COVID-19 or SARS-CoV-2 infection, including acute macular neuroretinopathy (AMN), optic neuritis (ON), neuroretinitis, retinal vascular occlusion, Purtschner like retinopathy, central serous retinopathy, papillophlebitis, optic neuritis, panuveitis, multifocal retinitis, and necrotizing retinitis, is rare (11). Here, we describe the case of a young woman with new-onset AMN after experiencing symptoms of COVID-19 infection. In addition, we reviewed and pooled available data from AMN patients following COVID-19 or SARS-CoV-2 infection.

## Methods

Patient signed informed consent forms. This study was conducted in accordance with the Declaration of Helsinki and approved by the Institutional Review Board of Chongqing Medical University, the First Affiliated Hospital of Chongqing Medical University (Approval No. 2023-181). A PubMed database search was performed for ‘COVID-19’, ‘SARS-CoV-2’, ‘ophthalmic manifestations’, ‘acute macular neuroretinopathy’, and ‘paracentral scotomas’. The reference lists of the obtained records were manually searched for additional reports. We included articles in the English language published between January 1, 2020, and March 31, 2023. There were no restrictions on study design, but duplicate reports were removed. The extracted data included patient demographic information, drug history, background conditions, COVID-19 or SARS-CoV-2 infection symptoms, infection-to-ocular symptom time intervals, symptom presentations, findings from imaging studies, treatment processes, and outcomes. While the search was not exhaustive, we tried to include all the articles.

## Results

### Case presentation

**Figure 1:**
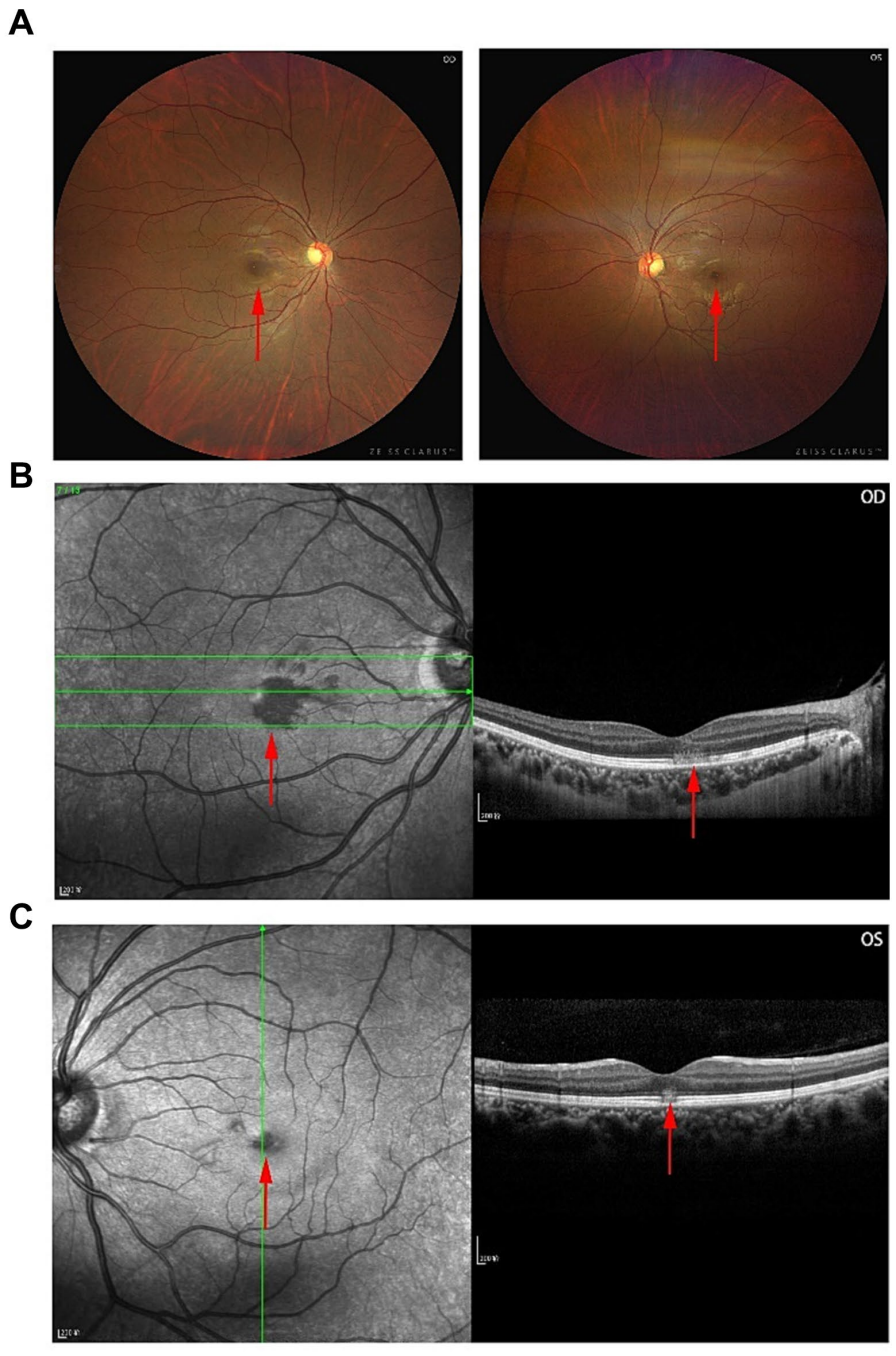
Multimodal images that display a partial reconstitution of the outer retinal architecture. **(A)** Fundus photographs of the right (OD) and left (OS) eyes at the time of presentation. **(B,C)** SD-OCT images of both eyes.

A 30-year-old Han woman complained of blurred vision in both eyes (more evident in the right eye) one day after the symptoms of COVID-19 or SARS-CoV-2 infection, i.e., fever (39.1°C), first appeared. The infection was diagnosed by reverse transcriptase polymerase chain reaction (PCR). Her visual acuity was 20/2000 OD and 20/32 OS. No relative afferent pupillary defect (RAPD) was found. No anterior segment abnormalities were detected. Color fundus imaging demonstrated perifoveal reddish-brown lesions OUs (Figure 1A). Near-infrared reflectance (NIR) imaging in both eyes revealed a well-demarcated, hyporeflective, oval-shaped macular lesion involving the fovea and that extended nasally, with the lesion area in the right eye being approximately three times that in the left eye (Figures 1B,C). Cross-sectional spectral-domain OCT (SD-OCT) revealed outer plexiform layer (OPL) thickening, outer nuclear layer (ONL) thinning, and disruption of the ellipsoid zone (EZ) in areas corresponding to the lesions (OU) (Figures 1B,C). She had no known ocular history, or systemic condition, and had not sought treatment prior to this presentation.

Given the acute development of these characteristic findings along with her clinical history, the patient was diagnosed with AMN. She was started on oral prednisolone 30 mg/day for 7 days. Afterward, the dose was reduced to 10 mg per week until 5 mg/day, after which the treatment was stopped. Notably, by one month, her visual acuity was 20/20 OD and 20/20 OS. During the four-month follow-up period, the patient’s visual acuity stabilized at 20/20, and no further discomfort was reported in either eye.

### Literature search results

In the literature, we found 19 articles reporting cases of AMN in people with recent COVID-19 or SARS-CoV-2 infection (see Table 1).

**Table 1:** Cases of AMN associated with COVID-19 or SARS-CoV-2 infection.

Case No./Author	Age/sex	Background illness/drug history	COVID-19 or SARS-CoV-2 manifestation	Interval time^*^	Presenting symptoms	Imaging features	Treatment	Outcome
1. Virgo and Mohamed (12)	37/F	Pregnancy/no	Cough, fever, anosmia	35 days	OS faintly colorful, paracentral scotoma, 20/20	OCT: hyperreflective change in IPL and OPL, INL volume loss	Unstated	Unstated
2. Virgo and Mohamed (12)	32/M	Acephalgic visual migraine aura/no	Unstated	16 days	OD faintly colorful, paracentral scotoma, 20/20	OCT: faint OPL hyperreflective change, IZ disruption	Unstated	Unstated
3. Gascon et al. (13)	53/M	Splenectomy /blind in OD due to traumatic glaucoma	Mild thoracic pain related to respiratory movements	8 days	OS negative scotoma, dyschromatopsia, 20/63	CFP: deep retinal hemorrhages, Roth spots; NIR: subtle, hyporeflective area, oval-shaped lesion surrounding the fovea; OCT: hyperreflectivity of the OPL, HFL and ONL, attenuation of EZ/IZ	Observation	2 weeks/ 20/32, partially resolved
4. Zamani et al. (14)	20/F	Acute myeloid leukemia/ chemotherapy	Dyspnea, malaise, cough	5 days	OD paracentral visual field defect and photopsia, 20/20	CFP: hemorrhages and Roth’s spots; NIR: hyperreflective patch; OCT: hyperreflectivity of the ONL and OPL	Unstated	Deceased after 6 days because of severe pneumonia
5. Aidar et al. (15)	71/F	Arterial hypertension and a kidney transplant due to hepatitis C/no	Fever, anosmia, dysgeusia, dyspnoea, and adynamia	2 weeks	OS low visual acuity; 0.5 LogMAR	CFP: foveal pigment mobilization, FFA: hypofluorescent fovea surrounded by irregular hyperfluorescent defects; OCT: central foveal thinning, EZ/IZ disrupted	Observation	2-month/no improvement
6. David and Fivgas (16)	22/F	Attention deficit disorder/lisdexamfetamine dimesylate; norgestimate and ethinyl estradiol	Headache	Unstated	OU ring of black dots with a wave in the middle; 20/20	CFP: multiple subtle reddish-brown petaloid lesions radiating from the fovea; OCT: disruption of OPL and ONL; attenuated reflectivity of EZ	Observation	6-month follow-up /slightly improved
7. El Matri et al. (17)	75/F	Diabetic/unstated	Unsated	A month	OD relative paracentral scotoma	CFP: non proliferative diabetic retinopathy; NIR: slightly hyporeflective lesion; OCT: a large hyperreflective band involving ONL and OPL, a fragmented EZ	Observation	Unstated
8. Masjedi et al. (18)	29/F	Unremarkable/no	Fever, headache, and cough	2 weeks	OS acute onset paracentral visual field defect	CFP: a yellow spot; NIF: a grayish wedge-shaped lesion with the hypo-reflective area; OCT: EZ disruption	Observation	2 months/ partially resolved
9. Mace and Pipelart (19)	39/F	Unremarkable/no	Cough and fever	2 days	OU photopsia and bilateral paracentral scotoma; 10/10	NIR: a bilateral grayish perifoveolar petaloids lesions; OCT: OPL hyperreflectivity	Observation	1 month/ symptoms persisted
10. Capuano et al. (20)	27/M	Unremarkable/no	No	Unstated	OS unilateral dyschromatopsia and paracentral scotoma; 20/20	CFP: a subtle yellowish perifoveal halo; OCT: hyperreflective lesions; OCTA: DCP hypoperfusion	Observation	2 weeks/ partially resolved
11. Capuano et al. (20)	37/F	Unremarkable/no	No	Unstated	OU paracentral scotomas; 20/20	OCT: OPL and ONL hyperreflective infarction, IS/OS and OS/RPE disruption	Observation	1 month/ partially resolved
12. Preti et al. (21)	70/M	Unremarkable/no	Fever, cough, vomiting, diarrhea, headache, and loss of taste	1 day	OS paracentral scotoma, 20/100	OCT: ONL hyperreflective, EZ disruption	Levofloxacin, azithromycin, and corticosteroids for 5 days	1 month/ resolved, 20/30 OS
13.Strzalkowski et al. (22)	18/F	Unremarkable/no	Headache, dizziness	Unstated	Central scotomas ou 20/20	CFP: discreetly altered reflex pattern; NIR: hyporeflective superficial petalloid lesions; OCTA: flux reduction in the choriocapillary	Observation	1 month/ partially resolved
14.Kovalchuk et al. (23)	16/F	Unremarkable/no	A mild course	1 day	OU aracentric scotomas 0.8 OD/0.63 OS	CFP: graybrownish petaloid perifoveal lesions; NIR: petaloid perifoveal lesions; OCT: interruptions of macular EZ; OCTA: decreased flow signals	Observation	1 month/ slightly improved
15. Hawley and Han (24)	21/F	Unremarkable/no	No	2 days	OU several small, bilateral paracentral scotomas (blind spots)	CFP: several discrete, reddish-brown ellipsoid lesions; NIR: hyporeflectivity OCT: heterogenous, hyperreflective thickening of the outer retina	Observation	Slow resolution
16. Bellur et al. (25)	64/F	Hypertension, deep vein thrombosis on dabigatran a 30-pack year smoking history	Vomiting and diarrhea	3 days	OU acute, persistent and central vision loss, 20/200	NIR: fairly well demarcated, oval, hyporeflective lesions; OCT: ONL thinning and EZ disruption; OCTA: flow voids in DCP and choriocapillaris	Oral prednisone 60 mg daily and tapered over 3 weeks	2 months/ mildly improved
17. Giacuzzo et al. (26)	23/F	Unremarkable/no	Fatigue, nasal congestion, headache, vertigo and sweating	2 weeks	OU several paracentral scotomas; 20/20	NIR: large, bilateral confluent hyporeflective lesions and smaller petaloid-shaped lesions; OCT: EZ/IZ disruption, ONL hyperreflectivity	Observation	1 month/no obvious change
18. Jalink and Bronkhorst (27)	21/F	Oral contraceptives	Unstated	4 weeks	OD scotoma temporal to the center and photopsias	CFP: a round, brown spot nasal to the fovea; OCT: OPL and ONL irregularities	Stop contraceptives and observation	3 months/ ongoing
19. Sanjay et al. (28)	25/F	β Thalassemia Trait, OD traumatic uveitis	Fever, headache, myalgia	3 days	OU blurring of vision and a shadow, 20/20	OCT: hyper-reflectivity at the level of outer plexiform and outer nuclear layer	Observation	1 month/ resolution

^*^Timing refers to the ophthalmologic onset of symptoms relative to positive COVID-19 symptoms. CFP, color fundus photography; NIR, near infrared; OCT, optical coherence tomography; OCTA, optical coherence tomography angiography; FFA, fluorescein angiography; DCP, deep capillary plexus; HFL, Henle fiber layer; INL, inner nuclear layer; IPL, inner plexiform layer; OPL, outer plexiform layer; ONL, outer nuclear layer; EZ, ellipsoid zone; EZ/IZ, ellipsoid and interdigitation zones.

## Discussion

At present, COVID-19 or SARS-CoV-2 infection can be asymptomatic or cause mild influenza-like symptoms, and severe cases can present with respiratory distress and multiple organ failure. COVID-19 or SARS-CoV-2 seems to employ mechanisms for receptor recognition. It can bind with angiotensin-converting enzyme 2 (ACE-2) with the assistance of transmembrane serine protein 2 (TMPRSS2) or enter host cells by binding with the CD147 spike protein, thereby triggering a series of symptoms (29). ACE-2 receptors are present in the retinal ganglion cell layer, inner plexiform layer, inner nuclear layer, and outer photoreceptor segments of the eye. Moreover, TMPRSS2 is expressed in multiple retinal neuronal cells, vascular and perivascular cells, and retinal Müller glial cells. SARS-CoV-2 RNA was found in the retinas of patients who died from COVID-19, suggesting viral entry into retinal cells (30). Endothelial damage and microthrombi are the main pathological changes that lead to ocular disease.

Ophthalmologists worldwide have reported various manifestations of infection in the eye. Ophthalmic images vary in terms of presentation, severity, and timing (31). COVID-19 or SARS-CoV-2 can directly cause damage via keratoconjunctivitis, epiphora, or chemosis. Hyperinflammation with cytokine storms, stasis with hypoxia, and stasis with hypoxia that activate coagulation mechanisms can cause retinal disease (7, 8, 31, 32). Elevated D-dimer, serum ferritin, and lactate dehydrogenase levels and increased ESR/CRP inflammatory marker levels are observed in patients with ocular manifestations even after recovering from COVID-19 (33).

AMN was first reported by BOS in 1975 (34). Since the outbreak of COVID-19 or SARS-CoV-2, the incidence of AMN has increased from 0.66/100,000 in 2019 to 8.97/100,000 in 2020 (*p* = 0.001) at Rothschild Foundation Hospital, Paris, France. It is more common in young people (aged 12–65, median age 26), with a male-to-female ratio of approximately 1: 4–6. It can affect both eyes and is characterized by photophobia, paracentral scotoma (72–100%), floaters (3%), and visual distortions (35). Possible risk factors for AMN include infection or febrile illness (47.5%), oral contraceptives (35.6%), the use of adrenaline (7.9%), severe nonocular trauma (5.9%), shock (5%), dehydration, preeclampsia, postpartum hypotension, ulcerative colitis, Behcet’s disease, systemic lupus erythematosus, leukemia, and vaccine-related complications. Microvascular ischemia of the choriocapillaris after COVID-19 or SARS-CoV-2 infection may lead to hypoxic insult to the middle and outer retinal layers.

According to the available literature, symptoms of AMN can arise 1 day to 1 month after COVID-19 or SARS-CoV-2 infection. Risk factors such as contraceptive pills should be avoided. Oral prednisone may be an effective treatment for those experiencing marked vision loss. It is crucial to conduct additional research to uncover a potential cause-and-effect relationship between AMN and COVID-19 or SARS-CoV-2. However, whether a genetic susceptibility exists is unknown. To reinforce this hypothesis, further investigations with a larger sample size, including individuals with and without ocular symptoms and incorporating prolonged follow-up times are needed. As the pandemic continues and vaccination programs are rolled out extensively, the number of AMN cases may increase. Ophthalmologists should remain vigilant about this disease, notably because patient characteristics may deviate from the norm.

## Data availability statement

The raw data supporting the conclusions of this article will be made available by the authors, without undue reservation.

## Ethics statement

The studies involving humans were approved by the Institutional Review Board of Chongqing Medical University, the First Affiliated Hospital of Chongqing Medical University. The studies were conducted in accordance with the local legislation and institutional requirements. The participants provided their written informed consent to participate in this study. Written informed consent was obtained from the individual(s) for the publication of any potentially identifiable images or data included in this article (Approval No. 2023-181).

## Author contributions

XW: Conceptualization, Data curation, Investigation, Methodology, Writing – original draft, Writing – review & editing. PW: Conceptualization, Investigation, Project administration, Writing – review & editing. JL: Data curation, Formal analysis, Writing – original draft. HJ: Data curation, Methodology, Validation, Writing – original draft. HX: Conceptualization, Formal analysis, Resources, Writing – original draft. HP: Investigation, Project administration, Supervision, Validation, Visualization, Writing – review & editing.

## References

[1] Hu B Guo H Zhou P Shi ZL. Characteristics of SARS-CoV-2 and COVID-19. Nat Rev Microbiol. (2021) 19:141–54. doi: 10.1038/s41579-020-00459-7, PMID: 33024307 PMC7537588

[2] Carabelli AM Peacock TP Thorne LG Harvey WT Hughes J Consortium C-GU. SARS-CoV-2 variant biology: immune escape, transmission and fitness. Nat Rev Microbiol. (2023) 21:162–77. doi: 10.1038/s41579-022-00841-7, PMID: 36653446 PMC9847462

[3] Francois J Collery AS Hayek G Sot M Zaidi M Lhuillier L. Coronavirus disease 2019-associated ocular neuropathy with Panuveitis: a case report. JAMA Ophthalmol. (2021) 139:247–9. doi: 10.1001/jamaophthalmol.2020.5695, PMID: 33331870

[4] Hutama SA Alkaff FF Intan RE Maharani CD Indriaswati L Zuhria I. Recurrent keratoconjunctivitis as the sole manifestation of COVID-19 infection: a case report. Eur J Ophthalmol. (2022) 32:NP17–21. doi: 10.1177/11206721211006583, PMID: 33781126 PMC9294609

[5] Otaif W Al Somali AI Al Habash A. Episcleritis as a possible presenting sign of the novel coronavirus disease: a case report. Am J Ophthalmol Case Rep. (2020) 20:100917. doi: 10.1016/j.ajoc.2020.100917, PMID: 32923742 PMC7476899

[6] Wu P Duan F Luo C Liu Q Qu X Liang L. Characteristics of ocular findings of patients with coronavirus disease 2019 (COVID-19) in Hubei Province, China. JAMA Ophthalmol. (2020) 138:575–8. doi: 10.1001/jamaophthalmol.2020.1291, PMID: 32232433 PMC7110919

[7] Uzun A Keles Sahin A Bektas O. A unique case of branch retinal artery occlusion associated with a relatively mild coronavirus disease 2019. Ocul Immunol Inflamm. (2021) 29:715–8. doi: 10.1080/09273948.2021.1933071, PMID: 34252339

[8] Yeo S Kim H Lee J Yi J Chung YR. Retinal vascular occlusions in COVID-19 infection and vaccination: a literature review. Graefes Arch Clin Exp Ophthalmol. (2023) 261:1793–808. doi: 10.1007/s00417-022-05953-7, PMID: 36598554 PMC9811047

[9] Tisdale AK Chwalisz BK. Neuro-ophthalmic manifestations of coronavirus disease 19. Curr Opin Ophthalmol. (2020) 31:489–94. doi: 10.1097/ICU.0000000000000707 33009081

[10] Tisdale AK Dinkin M Chwalisz BK. Afferent and efferent neuro-ophthalmic complications of coronavirus disease 19. J Neuroophthalmol. (2021) 41:154–65. doi: 10.1097/WNO.0000000000001276, PMID: 33935220

[11] Sanjay S Agrawal S Jayadev C Kawali A Gowda PB Shetty R. Posterior segment manifestations and imaging features post-COVID-19. Med Hypothesis Discov Innov Ophthalmol. (2021) 10:95–106. doi: 10.51329/mehdiophthal1427, PMID: 37641707 PMC10460223

[12] Virgo J Mohamed M. Paracentral acute middle maculopathy and acute macular neuroretinopathy following SARS-CoV-2 infection. Eye (Lond). (2020) 34:2352–3. doi: 10.1038/s41433-020-1069-8, PMID: 32620843 PMC7333786

[13] Gascon P Briantais A Bertrand E Ramtohul P Comet A Beylerian M. Covid-19-associated retinopathy: a case report. Ocul Immunol Inflamm. (2020) 28:1293–7. doi: 10.1080/09273948.2020.1825751, PMID: 33021856

[14] Zamani G Ataei Azimi S Aminizadeh A Shams Abadi E Kamandi M Mortazi H. Acute macular neuroretinopathy in a patient with acute myeloid leukemia and deceased by COVID-19: a case report. J Ophthalmic Inflamm Infect. (2021) 10:39. doi: 10.1186/s12348-020-00231-1, PMID: 33415590 PMC7790518

[15] Aidar MN Gomes TM de Almeida MZH de Andrade EP Serracarbassa PD. Low visual acuity due to acute macular neuroretinopathy associated with COVID-19: a case report. Am J Case Rep. (2021) 22:e931169. doi: 10.12659/AJCR.931169, PMID: 33930011 PMC8097744

[16] David JA Fivgas GD. Acute macular neuroretinopathy associated with COVID-19 infection. Am J Ophthalmol Case Rep. (2021) 24:101232. doi: 10.1016/j.ajoc.2021.101232, PMID: 34778601 PMC8577875

[17] El Matri K Werda S Chebil A Falfoul Y Hassairi A Bouraoui R. Acute macular outer retinopathy as a presumed manifestation of COVID-19. J Fr Ophtalmol. (2021) 44:1274–7. doi: 10.1016/j.jfo.2021.06.002, PMID: 34275662 PMC8264579

[18] Masjedi M Pourazizi M Hosseini NS. Acute macular neuroretinopathy as a manifestation of coronavirus disease 2019: a case report. Clin Case Rep. (2021) 9:e04976. doi: 10.1002/ccr3.4976, PMID: 34703607 PMC8521194

[19] Mace T Pipelart V. Acute macular neuroretinopathy and SARS-CoV-2 infection: case report. J Fr Ophtalmol. (2021) 44:e519–21. doi: 10.1016/j.jfo.2021.07.004, PMID: 34625310 PMC8426190

[20] Capuano V Forte P Sacconi R Miere A Mehanna CJ Barone C. Querques G: acute macular neuroretinopathy as the first stage of SARS-CoV-2 infection. Eur J Ophthalmol. (2022) 33:NP105–11. doi: 10.1177/11206721221090697, PMID: 35360952 PMC8980851

[21] Preti RC Zacharias LC Cunha LP Monteiro MLR. Acute macular Neuroretinopathy as the presenting manifestation of Covid-19 infection. Retin Cases Brief Rep. (2022) 16:12–5. doi: 10.1097/ICB.0000000000001050, PMID: 34001764

[22] Strzalkowski P Steinberg JS Dithmar S. COVID-19-associated acute macular neuroretinopathy. Fortschr Ophthalmol. (2022) 120:767–70. doi: 10.1007/s00347-022-01704-5, PMID: 35943530 PMC9361229

[23] Kovalchuk B Kessler LJ Auffarth GU Mayer CS. Paracentral scotomas associated with COVID-19 infection. Fortschr Ophthalmol. (2022) 120:323–7. doi: 10.1007/s00347-022-01726-z, PMID: 36085528 PMC9462629

[24] Hawley L Han LS. Acute macular neuroretinopathy following COVID-19 infection. N Z Med J. (2022) 135:105–7. PMID: 36201735 10.26635/6965.5835

[25] Bellur S Zeleny A Patronas M Jiramongkolchai K Kodati S. Bilateral acute macular neuroretinopathy after COVID-19 vaccination and infection. Ocul Immunol Inflamm. (2022) 31:1222–5. doi: 10.1080/09273948.2022.2093753, PMID: 35914286

[26] Giacuzzo C Eandi CM Kawasaki A. Bilateral acute macular neuroretinopathy following COVID-19 infection. Acta Ophthalmol. (2022) 100:e611–2. doi: 10.1111/aos.14913, PMID: 34041854 PMC8222889

[27] Jalink MB Bronkhorst IHG. A sudden rise of patients with acute macular neuroretinopathy during the COVID-19 pandemic. Case Rep Ophthalmol. (2022) 13:96–103. doi: 10.1159/000522080, PMID: 35350236 PMC8921888

[28] Sanjay S Gadde SGK Kumar Yadav N Kawali A Gupta A Shetty R. Bilateral sequential acute macular Neuroretinopathy in an Asian Indian female with beta thalassemia trait following (Corona virus disease) COVID-19 vaccination and probable recent COVID infection – multimodal imaging study. Ocul Immunol Inflamm. (2022) 30:1222–7. doi: 10.1080/09273948.2022.2026978, PMID: 35050826

[29] Gupta A Madhavan MV Sehgal K Nair N Mahajan S Sehrawat TS. Extrapulmonary manifestations of COVID-19. Nat Med. (2020) 26:1017–32. doi: 10.1038/s41591-020-0968-3 32651579 PMC11972613

[30] Reinhold A Tzankov A Matter MS Mihic-Probst D Scholl HPN Meyer P. Ocular pathology and occasionally detectable intraocular severe acute respiratory syndrome Coronavirus-2 RNA in five fatal coronavirus Disease-19 cases. Ophthalmic Res. (2021) 64:785–92. doi: 10.1159/000514573, PMID: 33472206

[31] Sen M Honavar SG Sharma N Sachdev MS. COVID-19 and eye: a review of ophthalmic manifestations of COVID-19. Indian J Ophthalmol. (2021) 69:488–509. doi: 10.4103/ijo.IJO_297_21, PMID: 33595463 PMC7942063

[32] Mahjoub A Dlensi A Romdhane A Ben Abdesslem N Mahjoub A Bachraoui C. Bilateral central serous chorioretinopathy post-COVID-19. J Fr Ophtalmol. (2021) 44:1484–90. doi: 10.1016/j.jfo.2021.10.001, PMID: 34756744 PMC8520853

[33] Sanjay S Bhakti Mistra S Patro SK Kawali A Shetty R Mahendradas P. Systemic markers in ophthalmic manifestations of post Corona virus Disease-19 (COVID-19). Ocul Immunol Inflamm. (2023) 31:410–5. doi: 10.1080/09273948.2021.2025253, PMID: 35138993

[34] Bos PJ Deutman AF. Acute macular neuroretinopathy. Am J Ophthalmol. (1975) 80:573–84. doi: 10.1016/0002-9394(75)90387-6 1180301

[35] Azar G Bonnin S Vasseur V Faure C Salviat F Clermont CV. Did the COVID-19 pandemic increase the incidence of acute macular Neuroretinopathy? J Clin Med. (2021) 10:5038. doi: 10.3390/jcm10215038, PMID: 34768555 PMC8585041

